# What affects the direct economic burden of non-communicable diseases on middle-aged and older adult people in Shaanxi Province?

**DOI:** 10.3389/fpubh.2023.1219199

**Published:** 2023-12-22

**Authors:** Xiaowei Yang, Ju’e Yan, Sha Lai, Chi Shen, Ning Duan

**Affiliations:** ^1^School of Public Policy and Administration, Xi’an Jiaotong University, Xi’an, Shaanxi, China; ^2^Honghui Hospital, Xi’an Jiaotong University, Xi’an, Shaanxi, China

**Keywords:** middle-aged and older adult people, non-communicable diseases, direct economic burden, pooled cross-sections regression model, effect factors

## Abstract

Non-communicable diseases (NCDs) are the leading cause of death worldwide. NCDs affect the health status and the quality of life. In addition, continuous NCDs treatment expenses place a heavy economic burden on families and cause huge economic losses to the society. The prevention and treatment of NCDs and reduction of their economic burden are key public health issues. Considering middle-aged and older adult people as the focus, their basic socio-demographic characteristics and health behavior status of this group, and a pooled cross-sections regression model was then used to analyze the main factors affecting the direct economic burden. The results showed that from 2013 to 2018, the prevalence of NCDs among the middle-aged and older adult people in Shaanxi province as well as the direct economic burden of NCDs increased. The effect factors primarily included sex, age, employment status, income level, type of medical insurance, urban or rural residency, level of the health care-providing institutions, visiting times of 2-week, and length of hospital stay. Several measures can be taken to control the onset of NCDs and reduce their direct economic burden.

## 1 Introduction

Non-communicable diseases (NCDs) that may be caused by genetic or behavioral factors and generally have a slow progression and long duration (1). Identified as “one of the major challenges for sustainable development in the twenty-first century” (2), NCDs are not a new problem and have pose an increasing threat to public health worldwide NCDs kill 41 million people each year, equivalent to 74.0% of all deaths globally (3). Cardiovascular diseases, cancer, chronic respiratory diseases, and diabetes account for more than 80% of these deaths (1). The growing burden of NCDs also exacts an economic cost, as people are less productive, work for fewer years, and die prematurely. Disability caused by NCDs has economic consequences at multiple levels: individual, household, economic agents, public institutions, government and the society as a whole (4). Moreover, the loss of labor and high cost of NCDs treatment seriously affect the coordination and sustainability of economic development (5).

The mortality rate of NCDs is higher in countries and regions with relatively lower economic development level. In the Global Status Report on NCDs (2014), the World Health Organization (WHO) indicated that 74.0% of NCDs-related deaths and 82.0% of premature deaths worldwide came from lower- and middle-income countries (LMICs) (6). If they continue their upward trend, NCDs were projected to cause a cumulative economic loss of up to US $7 trillion in LMICs by 2025 (7). In 2011, 2014 and 2018, the United Nations (U.N.) respectively held High-level Meeting on the Prevention and Control of NCDs, which led to great global attention to NCDs and call for global targets and an action plan for NCDs (8–11). Adopted in 2015 by all member-states of the U.N., the Sustainable Development Goals (SDGs) include an NCD target and several risk-factor-related targets for achievement by 2030.According to the Report on Nutrition and NCDs in China (2020), from 2005 to 2015, the cumulative economic losses caused by cardiovascular diseases, stroke, and diabetes were estimated to reach $550 billion (12). If prevention and control measures are not taken in time, the losses may continue to climb at an even faster rate. At present, the total number of Chinese residents suffering from NCDs is approximately 180 million, and the economic burden caused by NCDs accounts for >70.0% of the total health expenditure (13), of which 50.0% is borne by people aged >65 years. The seventh national census in China showed that the number of people aged >60 years was 264.02 million, accounting for 18.7% of the entire population (the number of those aged >65 years was 19.06 million, i.e., 13.5% of the entire population). Furthermore, population aging is deepening. As chronic course, NCDs can lead long-term economic consequence for individuals and their households. This economic burden also affects health through reduce abandonment or interruption of therapy and impaired quality of life (14). Taken together, optimizing NCDs prevention and control means and reducing family economic burden caused by NCDs treatment are of great significance for promoting social stability and sound development.

Many studies focused on the economic effects of NCDs on households. Some of these studies (15–17) discussed the direct out-of-pocket expenses caused by NCDs, while the others (18, 19) discussed financial effects, such as loss of work and coping strategies. To reduce the economic burden of NCDs, it is important to design the financial protection programs to avert illness-related economic hardship and encourage access to therapy. Thus for whom, their prevalence of NCDs and direct economic burden are taken into account for policy making. Most studies were cross-sectional in design. Consequently the results could be imprecise. So the data or analysis method should be improved.

The present study aimed to explore the prevalence of NCDs in middle-aged and older adult people and changes in patients’ direct economic burden. These data were analyzed the effect factors of direct economic burden and provided a scientific basis for formulating a policy to control the rise of expenditure for NCDs.

## 2 Methods

### 2.1 Data sources

Data were derived from National Health Services Survey(NHSS) conducted at five sample sites in Shaanxi province in 2013 and 2018. The multistage stratification cluster sampling method was used to sample participants from Jintai district, Mei county, Hanyin county, Lintong district, and Weicheng district. These 5 districts(counties) were stable sample sites for each NHSS which had been conducted every 5 years by the National Health Commission of the People’s Republic of China (NHPC) since 1993. First, 25 sub-districts(townships) were randomly selected in sampled districts(counties). Next, 50 communities(villages) were randomly selected from sub-districts(townships). Finally, 3,000 households were interviewed. All family members who lived at home for the past 6 months participated in the survey. Face-to-face inquiry was adopted in the survey, which included detailed information on family economic status, sociodemographic characteristics, health behaviors and health status, health service utilization, and health expenditure.

A series rigorous quality control measures were implemented during data collection. First, the interviewer were medical staffs from local communities or villages and were well trained before conducting the interviews. Second, an appointment was made prior to the survey to ensure that the respondents knew when the interview would take place. If the respondents were not at home, the interviewers would return for a maximum of three times to complete the survey. Third, the respondents were required to reply the questionary by themselves, except for children under 6 years old. Fourth, strict inspection was carried by supervisors, if there were missing information and logical errors in the questionary, a resurvey was required the next day. Moreover, 5% of the households were revisited, and eight questions were re-interviewed to check for consistency. Based on these efforts, high response rates (>85%) and high consistency between survey and re-interviewed survey (>95%) were achieved in both surveys. The self-response rates were also above 75% in both surveys.

The two surveys, respectively, surveyed 7,653 and 7,819 people. Shaanxi province had a population of 37.6 million in 2013 (48.7% lived in rural areas) and 38.6 million in 2018 (41.9% lived in rural areas). The Myer’s Blended Index was 1.63 in 2013 and 1.67 in 2018, indicating that the respondents were representative of Shaanxi Province for age in both surveys.

In this study NCD was defined as chronic diseases that were diagnosed by a doctor within 6 months before the survey. Or the patient was diagnosed with a chronic disease by a doctor 6 months before the survey, and had an attack in the last 6 months and had taken treatment measures such as medication, physiotherapy, or had been treated to control the attack of a chronic disease. These diseases or conditions included cerebrovascular and cardiovascular consequences of atherosclerosis, hypertension, diabetes mellitus, renal insufficiency, mental disorders, and cancer etc.

Considering that middle-aged and older adult people would bear a heavier economic burden of NCDs, people aged ≥45 years were selected as the middle-aged and older adults group for the present study as per the age classification standard of the WHO.

This study aimed to investigate the direct economic burden of NCDs. Thus, individuals aged ≥45 years who reported chronic diseases were included in the study. A total of 2,030 people in 2013 and 2,448 people in 2018 were finally included, with a total sample size of 4,478 people.

### 2.2 Research variables

#### 2.2.1 Direct economic burden of NCDs

The economic burden of NCDs can be classified as follows: direct economic burden, indirect economic burden, and intangible economic burden (20). Direct economic burden is a direct reflection of the cost that patients bear on receiving therapy. It mainly includes the direct medical expenses paid by patients during treatment (such as diagnosis, treatment, medicine, nursing, surgery, and medical consumable costs) as well as the direct nonmedical expenses of medical treatment (such as transportation, catering, and accommodation expenses) (21).

The direct economic burden of NCDs includes the expenditure of 2 weeks’ healthcare and annual hospitalization for NCDs. Medical expenses for 2 weeks of NCDs treatment refer to outpatient, self-treatment, and direct nonmedical expenses incurred by patients for NCDs treatment. Annual hospitalization expenses for NCDs refer to the expenses paid by patients for hospitalization for NCDs in the past year and the direct nonmedical expenses incurred by hospitalization. Expenditures reimbursed by insurance and insurance premiums were not included in the data analysis.

If the respondent reported chronic disease and the name of the illness within 2 weeks matched the name of the chronic disease record, it was defined as “the 2 weeks’ period of NCDs treatment.” The same as “the annual hospitalization for NCDs.”

#### 2.2.2 Control variables

The health expenditure of middle-aged and older adult patients with NCDs is related to their sociodemographic characteristics, health behavior, health status, and health service utilization (22). Therefore, the following variables that affect the direct economic burden of NCDs were studied: sex, age, education level, employment status, *per capita* annual income, marital status, urban or rural residency, number of permanent family residents, type of medical insurance, smoking status, alcohol consumption status, regular exercise, self-reported health status, overweight status, multiple chronic conditions(MCCs), level of the health care-providing institutions, frequency of visits in 2 weeks, length of hospital stay, surgical history, and others.

### 2.3 Data analysis

#### 2.3.1 General statistical methods

Descriptive statistics were used to analyze the sociodemographic characteristics, health behavior, and health status of the study group, and Pearson’s chi-square test was used to compare differences between the two surveys. The mean and median of the direct disease economic burden of NCDs were used to describe the central tendency. As the medical expense data were not normally distributed, the Wilcoxon signed-rank sum test was used to analyze changes in the cost of NCDs treatment.

#### 2.3.2 Pooled cross-sections regression model

A pooled cross-sections regression model was used to analyze the factors affecting the direct economic burden of NCDs. As no follow-up survey was conducted on the study group, multiple random sampling was performed in the same group and independent samples extracted at different time nodes were combined to form pooled cross-section data (23). When performing ordinary least squares regression, the data need to be corrected by adding time dummy variables.


$$y_{\mathrm{it}}=\beta_{0}+\delta_{k}D_{k}+\beta^{\prime }X_{\mathrm{it}}+\varepsilon_{\mathrm{it}}$$

Where i represents the unit number of cross section, t represents each observation during the period T, y is the dependent variable, X is a different argument, and ε is the error term. As the population will have different distributions in different periods, the above model enables the intercept to have different values in different years, which can be achieved by introducing dummy variables. In the given equation, D represents the dummy variable of time, k is the number of dummy variables [k should be no more than (T-1)]. β_0_ is the constant term and β′ is the coefficient of the independent variable.

#### 2.3.3 Discount

To make the medical expense data of the two surveys comparable, the data of 2013 were discounted according to the medical and health price consumption index, which was derived from the Health Statistical Yearbook of Shaanxi province. From year 2014 to 2018 the medical and health price consumption index in Shaanxi province were 102.7, 102.0, 102.4, 108.6 and 104.0%, respectively. If the medical expense in 2013 was Y_2013_, and Y_2013_ discounted in 2018 was Y_2018_, then,


$$Y_{2018}=Y_{2013}*102.7\%*102.0\%*102.4\%*108.6\%*104.0\%$$

## 3 Results

There were 4,661 middle-aged and older adult people in 2013 and the number of such people with NCDs was 2030, with the prevalence of NCDs being 43.55%. In 2018, 4,443 middle-aged and older adult people were included in the sample group and 2,448 of these had NCDs, with the NCD prevalence of 55.10%. Thus, the prevalence of NCDs in the middle-aged and older adult population significantly increased by 11.55% from 2013 to 2018 (χ^2^ = 56.032, *p* < 0.001).

### 3.1 Sociodemographic characteristics

Among the study population suffering from NCDs, the education level, occupational status, *per capita* annual income, urban or rural residency, and number of permanent family members were significantly different between the two health services surveys (Table 1). Compared with 2013, the proportion of patients with primary school education or below and those with employment significantly decreased by 5.46 and 15.15%, respectively, in 2018. Nevertheless, the proportion of patients with high-income and those living in urban areas significantly increased by 12.57 and 3.41%, respectively. In addition, the proportion of patients with three or less permanent family members significantly increased by 12.52%. The two primary social medical insurance schemes in China are the Urban Employee Medical Insurance (UEMI) and the Urban and Rural Resident Medical Insurance (URRMI). The Other Medical Insurance (OMI) encompasses any insurance scheme that is not included in the aforementioned two.

**Table 1:** Sociodemographic details of the study population.

Sociodemographic characteristics		2013 (*N* = 2030)	2018 (*N* = 2,448)	χ^2^	*p*
		Frequency, n(%)	Frequency, n(%)		
Sex	Male	950(46.80)	1,154(47.14)	0.05	0.820
	Female	1,080(53.20)	1,294(52.86)		
Age	45-	823 (40.54)	953(38.93)	3.00	0.223
	60-	905(44.58)	1,154(47.14)		
	≥75	302(14.88)	341(13.93)		
Education level	Primary school and below	963 (47.58)	1,031(42.12)	14.13	0.001
	Secondary and technical schools	965 (47.40)	1,260(51.47)		
	College or above	102 (5.02)	157(6.41)		
Marital status	Married	1,692 (83.35)	2078(84.89)	1.96	0.161
	Widowed, divorced	338(16.65)	370(15.11)		
Occupational status	Employment	1,048(51.63)	893(36.48)	129.47	<0.001
	Unemployment	427 (21.03)	978(39.95)		
	Retired	555(27.34)	577(23.57)		
*Per capita* annual income	Lower	431(21.23)	497(20.30)	142.53	<0.001
	Lower middle	449(22.12)	434 (17.73)		
	Middle	470(23.16)	402(16.42)		
	Upper middle	401(19.75)	471(19.24)		
	Upper	279 (13.74)	644(26.31)		
Insurance	URRMI	1,322(65.12)	1,627(66.46)	0.76	0.411
	UEMI	573(28.23)	668(27.29)		
	OMI	135 (6.65)	153(6.25)		
Urban or rural residency	Rural	867 (42.71)	962(39.30)	5.35	0.021
	Urban	1,163 (57.29)	1,486(60.70)		
Number of permanent family residents	≤3	1,402(69.06)	1997(81.58)	95.01	<0.001
	>3	628 (30.94)	451(18.42)		

### 3.2 Behavior and health status

Table 2 showed the behavior and health status of the study population. The proportion of smokers and alcohol consumers significantly increased from 23.84 to 28.19% and 15.91 to 21.73%, respectively. The proportion of people who exercised regularly significantly decreased from 60.25 to 43.83% and that of persons who had undergone a health examination significantly increased from 50.34 to 53.23% in the latest year. The proportion of patients who were overweight and those with MCCs significantly increased from 23.15 and 27.04% to 38.56 and 31.13%, respectively. Moreover, the self-reported health status of patients became worse. Indeed, the proportion of patients with scores <70 significantly increased from 27.00 to 40.85%, whereas that of patients with scores >85 significantly decreased from 23.15 to 13.81%. Self-reported health status is one dimension of EuroQoL five-dimension questionnaire (EQ-5D), which could assess individual health status (24).

**Table 2:** Behavior and health status of the study population.

Variable		2013 (*N* = 2030)	2018 (*N* = 2,448)	χ^2^	*p*
		Frequency, n(%)	Frequency, n (%)		
Smoker	No	1,546(76.16)	1758(71.81)	10.83	0.001
	Yes	484 (23.84)	690(28.19)		
Alcohol consumer	No	1707 (84.09)	1916(78.27)	24.34	<0.001
	Yes	323 (15.91)	532(21.73)		
Exercised regularly	No	807 (39.75)	1,375(56.17)	188.52	<0.001
	Yes	1,223 (60.25)	1,073(43.83)		
Health examination	No	1,008(49.66)	1,145(46.77)	5.67	0.017
	Yes	1,022 (50.34)	1,303(53.23)		
Overweight	No	1,560 (76.85)	1,504(61.44)	7.19	0.007
	Yes	470 (23.15)	944(38.56)		
Chronic disease comorbidities	No	1,481(72.96)	1,686(68.87)	13.57	0.001
	Yes	549 (27.04)	762(31.13)		
Self-reported health status	<70	548 (27.00)	1,000(40.85)	120.10	<0.001
	70-	1,012(49.85)	1,110(45.34)		
	≥85	470(23.15)	338(13.81)		

### 3.3 Direct economic burden

To investigate the direct disease economic burden on the study population in 2013 and 2018, the 2-week medical expenses and annual inpatient medical expenses of the two sample populations were calculated. The medical expenses in 2013 were discounted by the medical and health price consumption index. As shown in Table 3, the mean and median of 2-week medical expenses in the two surveys were 262.6 RMB and 127.2 RMB, and 583.1 RMB and 220.0 RMB, respectively. The median values analyzed using Wilcoxon signed-rank sum tests were significantly different, indicating that the 2-week medical expenses in 2018 were higher than those in 2013. The mean and median annual inpatient medical expenses were 3650.6 RMB and 2301.9 RMB, and 4914.2 RMB and 2450.0 RMB, respectively. The median values analyzed via Wilcoxon signed-rank sum test were not significantly different, indicating that the annual inpatient medical expenses were similar.

**Table 3:** Direct economic burden *per capita* of the study population.

Health service utilization	Year	Patient	Mean			Median	Z	*p*
			Direct medical expenses (RMB)	Direct non-medical expenses(RMB)	Total (RMB)			
2-week	2013	579	240.3	22.3	262.6	127.2	37.40	<0.001
	2018	617	521.9	61.2	583.1	220.0		
Annual admission	2013	566	3040.5	610.1	3650.6	2301.9	0.13	0.772
	2018	664	4141.8	772.4	4914.2	2450.0		

### 3.4 Effect factors of direct economic burden

After discounting the 2-week medical expenses and annual inpatient medical expenses in 2013 using the medical and health consumer price index, the data of 2018 were combined. To avoid the impact of right-skewed distribution characteristics of medical expense data on regression analysis, the medical expense data were logarithmically converted before pooled cross-sections regression analysis to ensure the accuracy of results.

Individual adjusted regressions model for 2013 and 2018 were first performed on the factors affecting the 2-week medical expenses and annual inpatient medical expenses of NCDs. And then the pooled cross-sections regression model was made for analysis.

Table 4 showed determinants of OOP for 2-week medical expenses. For the result of pooled cross-sections regression, the age, urban or rural residency and self-reported health status were significantly negatively correlated with the 2-week medical expenses for NCDs. These results indicated that the medical expenses of patients aged >60 years were lower than those of middle-aged patients and that the overall payment level of urban residents was lower than that of rural residents. Moreover, with the increase in self-reported health scores, the 2-week expenses showed an obvious decline. The year, employment status, UEMI, MCCs, health care-providing institutions, and visiting times were significantly positively correlated with the 2-week medical expenses for NCDs. Thus, compared with actively working patients, the expenses of unemployed and retired patients were higher. Further, the expenses of patients having UEMI and those with NCD MCCs were higher than the expenses of patients having URRMI and those with only one NCD. With the increase in the level of the health care-providing institutions and improvement in the utilization rate of outpatient service, the medical expenditure also increased significantly.

**Table 4:** Determinants of OOP for 2-week medical expenses.

	2013			2018			combined 2013 and 2018		
Variables	Coefficient	Standard error	*t*	Coefficient	Standard error	*t*	Coefficient	Standard error	*t*
Year									
2013	_	_	_	_	_	_	Ref		
2018	_	_	_	_	_	_	0.23	0.08	2.96*
Sex									
Female	Ref			Ref			Ref		
Male	−0.02	0.13	−0.18	0.19	0.14	1.41	0.10	0.10	1.09
Age									
45-	Ref						Ref		
60-	−0.16	0.14	−1.19	−0.29	0.12	−2.36*	−0.24	0.09	−2.71*
≥75	−0.10	0.19	−0.53	0.03	0.19	0.18	−0.05	0.14	−0.36
Education level									
Primary school and below	Ref			Ref			Ref		
Secondary and technical schools	0.14	0.13	1.08	0.10	0.12	0.85	0.12	0.09	1.43
College or above	−0.19	0.28	−0.66	−0.04	0.22	−0.17	−0.07	0.17	−0.40
Marital status									
Married	Ref			Ref			Ref		
Widowed, divorced	−0.23	0.15	−1.52	−0.08	0.15	−0.56	−0.13	0.11	−1.20
Occupational status									
Employment	Ref						Ref		
Unemployment	0.25	0.14	1.81	0.30	0.12	2.56*	0.31	0.09	3.40*
Retired	0.19	0.20	0.92	0.48	0.22	2.14*	0.29	0.15	1.87
*Per capita* annual income									
Lower	Ref			Ref			Ref		
Lower middle	−0.14	0.15	−0.91	−0.01	0.16	−0.01	−0.06	0.11	−0.51
Middle	−0.10	0.16	−0.67	0.21	0.15	1.38	0.08	0.11	0.76
Upper middle	0.06	0.18	0.33	0.11	0.15	0.70	0.08	0.11	0.71
Upper	−0.02	0.23	−0.07	0.08	0.17	0.47	0.07	0.13	0.52
Urban or rural residency									
Rural	Ref			Ref			Ref		
Urban	−0.10	0.13	−0.82	−0.33	0.11	2.94*	−0.25	0.08	−3.00*
Medical Insurance									
URRMI	Ref			Ref			Ref		
UEMI	o.76	0.21	3.60*	0.38	0.23	1.66	0.57	0.16	3.60*
OMI	0.17	0.26	0.65	0.03	0.16	0.16	0.05	0.13	0.37
Self-reported health status									
<70	Ref			Ref			Ref		
70-	−0.37	0.12	−3.05*	−0.22	0.11	−2.02*	−0.28	0.08	−3.46*
≥85	−0.38	0.16	−2.40*	−0.67	0.17	−3.95*	−0.50	0.12	−4.36*
MCCs									
No	Ref			Ref			Ref		
Yes	0.07	0.11	0.60	0.30	0.11	2.74*	0.19	0.08	2.41*
Healthcare providing institutions									
Township	Ref			Ref			Ref		
County/District	1.30	0.15	8.87*	1.36	0.11	11.85*	1.33	0.09	14.96*
Province/City	1.64	0.30	5.50*	2.41	0.30	8.14*	2.07	0.21	9.88*
Visiting times									
1	Ref			Ref			Ref		
≥2	1.15	0.11	10.42*	1.20	0.10	11.57*	1.17	0.08	15.40*
Constant	4.06	0.26	15.75*	4.61	0.22	21.45*	4.27	0.17	24.72*
F	13.66*			17.53*			28.99*		
Adj R-squared	0.40			0.36			0.37		

*p < 0.05.

The significant determinants of OOP for 2-week medical expenses were different in 2013 and 2018. Compare with 2018, the age, employment status, urban or rural residency and MCCs in 2013 were not significant.

Table 5 showed determinants of OOP for annual inpatient medical expenses. For the result of pooled cross-sections regression, among the variables that significantly affected the expenditure of hospitalization, OMI and self-reported health status were negative. OMI could better reflect the role of reducing the amount of out-of-pocket expenses. The hospitalization expenses of urban residents were significantly higher than those of rural residents. Compare with 2018, the sex, urban or rural residency were significant, while employment status, medical insurance, self-reported health status and MCCs in 2013 were not significant.

**Table 5:** Determinants of OOP for annual inpatient medical expenses.

	2013			2018			combined 2013 and 2018		
Variables	Coefficient	Standard error	*t*	Coefficient	Standard error	*t*	Coefficient	Standard error		*t*
Year										
2013	_	_	_	_	_	_	Ref			
2018	_	_	_	_	_	_	0.11	0.05	2.27*	
Sex										
Female	Ref			Ref			Ref			
Male	0.15	0.07	2.01*	0.11	0.08	1.41	0.11	0.05	2.08*	
Age										
45-	Ref						Ref			
60-	0.07	0.08	0.90	−0.02	0.08	−0.23	0.03	0.06	0.47	
≥75	0.10	0.11	0.87	−0.01	0.11	−0.07	0.03	0.08	0.39	
Education level										
Primary school and below	Ref			Ref			Ref			
Secondary and technical schools	−0.09	0.07	−1.29	0.02	0.08	0.24	−0.03	0.05	2.65*	
College or above	0.05	0.13	0.38	0.09	0.12	0.70	0.10	0.09	−0.64	
Marital status										
Married	Ref			Ref			Ref			
Widowed, divorced	−0.09	0.09	−1.08	−0.14	0.09	−1.55	−0.11	0.06	−1.72	
Occupational status										
Employment	Ref						Ref			
Unemployment	−0.01	0.09	−0.01	0.31	0.08	3.94*	0.15	0.06	2.65*	
Retired	0.10	0.12	−0.89	0.04	0.13	0.29	−0.06	0.09	−0.64	
*Per capita* annual income										
Lower	Ref			Ref			Ref			
Lower middle	0.01	0.09	0.11	0.02	0.10	0.24	0.02	0.07	0.30	
Middle	0.12	0.09	1.36	0.10	0.10	0.96	0.12	0.07	1.76	
Upper middle	0.22	0.10	2.17*	0.11	0.10	0.99	0.15	0.07	2.03*	
Upper	0.23	0.12	1.97*	0.10	0.10	0.96	0.22	0.08	2.86*	
Urban or rural residency										
Rural	Ref			Ref			Ref			
Urban	0.10	0.07	1.30	0.36	0.08	4.55*	0.22	0.05	4.16*	
Medical insurance										
URRMI	Ref			Ref			Ref			
UEMI	−0.25	0.12	−2.05*	−0.01	0.13	−0.01	−0.14	0.09	−1.57	
OMI	−0.32	0.13	−2.42*	−0.05	0.11	−0.40	−0.19	0.09	−2.17*	
Self-reported health status										
<70	Ref			Ref			Ref			
70-	−0.10	0.07	−1.45	−0.20	0.07	−2.93*	−0.14	0.05	−2.92*	
≥85	−0.16	0.10	−1.64	−0.32	0.10	−3.07*	−0.19	0.07	−2.72*	
MCCs										
No	Ref			Ref			Ref			
Yes	0.03	0.07	0.48	0.17	0.07	2.54*	0.11	0.05	2.41*	
Healthcare providing institutions										
Township	Ref			Ref			Ref			
County/District	1.25	0.08	15.44*	1.00	0.09	11.47*	1.13	0.06	18.89*	
Province/City	1.76	0.10	17.06*	1.77	0.13	14.14*	1.73	0.08	21.46*	
Length of hospital stay										
≤7	Ref			Ref			Ref			
8–14	0.46	0.08	5.75*	0.16	0.07	2.28*	0.27	0.05	5.09*	
>14	0.98	0.08	11.60*	0.77	0.09	8.62*	0.82	0.06	13.49*	
Surgical history										
No	Ref			Ref			Ref			
Yes	0.58	0.07	8.01*	0.67	0.09	8.28	0.62	0.05	11.32*	
Constant	5.87	0.16	37.29*	6.06	0.14	42.72*	5.92	0.11	54.77*	
F	30.39*			25.39*			48.99*			
Adj R-squared	0.56			0.47			0.49			

*p < 0.05.

## 4 Discussion

In 2018, the prevalence of NCDs in the sample group reached 55.10%, an increase of 11.55% over 2013. In the study population, the number of patients who smoked, consumed alcohol, and were overweight increased; number of patients who regularly exercise decreased; and self-reported health status became worse. The increase in the incidence of NCDs is associated with lifestyle changes (25, 26). It was proved that behavioral risk factors for NCDs include tobacco use, harmful use of alcohol, unhealthy diets and physical inactivity (27). The prevention of NCDs should first advocate a healthy lifestyle and reduction in the influence of health risk factors.

The proportion of middle-aged and older adult patients with NCDs aged >60 years has increased owing to population aging. Many studies have shown that the older adults have a greater demand for health services and a higher utilization rate of health services (28–30). Increase in the number of older adult people suffering from NCDs will further aggravate the economic burden of NCDs.

The *per capita* direct economic burden of NCDs in the sample population increased from 2,965.11 RMB (3,592.32 RMB after discount) to 5,153.45 RMB from 2013 to 2018, and the direct economic burden faced by patients’ families concomitantly increased. The disease status of middle-aged and older adult people in the sample area revealed that the proportion of NCDs and MCCs in the population increased, which was likely to improve the utilization rate of health services. A review showed that with the improvement in the utilization rate of health services and rising expenditure of diagnosis and treatment, some patients with MCCs were affected with a relatively heavy economic burden (31). NCDs require long-term treatment as they are characterized by repeated bouts, and the economic burden of common NCDs on patients is thus heavier.

The main factors affecting the direct economic burden of NCDs on middle-aged and older adult patients included gender, age, occupational status, income level, type of medical insurance, urban or rural residency, health care-providing institution, visiting times of 2-week illness, and length of hospital stay. In particular, the medical expenditure of male patients was higher than that of female patients, possibly because men, as the economic pillar of the family, receive more attention and receive treatment on priority compared with women (22).

There was a negative relationship between 2-week medical expenses and age. The treatment expenses of the 45–59-year-old group were significantly higher than those of the 60–74-year-old group. This may be because the 45–59 age group could work and was the breadwinner of the family, so they were more likely to seek treatment for quick health recovery during the initial symptoms. With increasing age, the inpatient medical expenses of patients with NCDs had an increasing trend. Unlike outpatients and self-treated patients, older inpatients tended to have more severe and complex medical conditions and their hospitalization costs were relatively high.

The medical expenditure of unemployed and retired patients was higher than that of employed people. This result is similar to that of the China Family Pension Tracking Survey in 2015, which was analyzed using regression analysis (32). This may be because the working population is more economically independent and able to invest in their health, thereby reducing the cost of medical treatment by purchasing supplementary medical insurance.

Another study has shown that the higher-income group might spend more on medical and health expenses (33). Similar results were also found in the current study. The results of the regression model revealed that the hospitalization costs of the upper middle-income group and the upper-income group increased significantly. To receive better treatment, high-income people will choose expensive health services, sometimes resulting in certain unnecessary expenditures. Patients with low ability to pay tend to be more cautious in diagnosis and treatment, try to avoid excessive costs to the family economic burden, and sometimes do not get hospitalized even when hospitalization is warranted.

The type of medical insurance also significantly affects the medical and health costs of middle-aged and older adult patients with NCDs. The higher level of 2-week medical expenditure of patients participating in UEMI may be because UEMI is paid by individual accounts, which improves the demand for health services and the utilization rate of outpatient services to some extent. In the regression analysis of hospitalization costs, the level of expenditure on health costs was lower in the OMI group. Perhaps because the reimbursement ratio of commercial medical insurance is higher than that of basic medical insurance, the OMI insured out-of-pocket payment of patients was lower in this study.

The 2-week regression analysis of NCD expenditure showed that the urban where patients lived was a factor that reduced medical costs. This is because the health literacy rate of urban residents is generally higher than that of rural residents (34). Moreover, urban residents have more health awareness and regulate their own health behaviors and many of them receive early detection and treatment of diseases. Thus, their symptoms are relatively mild during diagnosis and treatment and the medical expenses are relatively low. However, the regression analysis of hospitalization cost revealed the opposite result, i.e., the hospitalization cost of urban residents was significantly higher than that of rural patients. This may be because urban residents tend to pay more for hospitalization in large high-level urban medical institutions.

Regression analysis revealed that the level of medical institution selected by the study population had a significant impact on the direct disease economic burden. There are obvious cost differences among different levels of medical institutions. Regarding the payment of outpatient expenses, the *per capita* medical expenditure of provincial and municipal hospitals is 1.73 times and 1.38 times of that of county-level hospitals, respectively, and for hospitalization costs, the *per capita* expenditure of provincial and municipal hospitals is 3.49 times and 2.27 times of that of county-level hospitals, respectively (35). The hospitalization cost of NCDs increases exponentially with improvement in the hospital level (36).

The main feature of the study was to analyze the effect factors of direct disease economic burden of NCDs on the middle-aged and older adult patients using the pooled cross-sections regression model combined with temporal changes. These were not the same participants between 2013 and 2018 surveys. Because the two surveys both used a rigorous random sampling process, respondents were less likely to participate in both surveys. As previously mentioned, the representativeness of the sample in Shaanxi province could be guaranteed. The merits of the study are as follows. First, data from two health services surveys were combined to increase the sample size, making the analysis more accurate. Second, changes in the direct disease economic burden of NCDs on the middle-aged and older adult patients could be observed over time. However, some limitations should be noted. The study relied on self-reported health data, which can be subject to recall bias. However, in health services survey, some households provided institutional bill of health to the investigators to record medical expenditure more accurately. Although it was not possible to distinguish which respondents provided institutional bills because the survey did not record it. Otherwise proportion of sample presented institutional bills instead of self-reported economic data could be calculated. Finally, data from a single province were included. Owing to the different socioeconomic conditions and medical insurance reimbursement policies in different regions, the results of this study cannot be extrapolated.

## 5 Conclusion

The prevalence of NCDs among the middle-aged and older adult people in Shaanxi province increased along with the direct economic burden. To reduce the direct economic burden of NCDs, special attention should be paid to women, older adults, rural residents, and unemployed or retired patients, and supplementary medical insurance should be increased for these special groups. At the same time, the service capacity and quality of basic medical institutions should be improved and hierarchical diagnosis and treatment policy should be implemented to reduce medical expenditure.

## Data availability statement

The datasets presented in this article are not readily available because the data used in this study belong to the Health Commission of Shaanxi Province and contain the personal information (e.g., name, personal communication information, property status etc.) of participants. The authors were involved in data collection. Due to the sensitive nature of these data and restrictions imposed by the Health Commission of Shaanxi Province, the authors cannot make these data publicly available. Requests to access the datasets should be directed to Health Commission of Shaanxi Province, sxwjwwz@126.com.

## Ethics statement

Informed consent was obtained from household members before the interview. Approval for this study was obtained by the Ethics Committee of Health Science Center, Xi’an Jiaotong University (approval number 2020–1256).

## Author contributions

XY conceived of the study and participated in its design, data analysis and interpretation, and was the primary person responsible for drafting the manuscript. JY participated in study design, data analysis, writing, and revision. ND contributed to study design and reviews. SL and CS participated in reading the draft and provided comments. All authors contributed to the article and approved the submitted version.
